# In this month’s Bulletin

**DOI:** 10.2471/BLT.21.001121

**Published:** 2021-11-01

Elena Altieri et al. (754) introduce this month’s theme on the use of behavioural science in health. Tedros Adhanom Ghebreyesus (755) provides an overview of the ways in which behavioural science can be applied.

Gary Humphreys (758–759) reports on systemic approaches to safer roads. Joyce Wamoyi talks to Andréia Azevedo Soares (760–761) about attributes of behaviour change initiatives in sub-Saharan Africa.

## Ethiopia 

### Trachoma control

Solomon Aragie et al. (762–772) study ways to change hygiene behaviours. 

## India

### Normalizing better maternal health 

Rajiv N Rimal et al. (773–782) trial interventions to increase iron and folic acid consumption.

## Rwanda 

### Insights from systems thinking 

Catherine Decouttere et al. (783–794) explore vaccine hesitancy.

## South Africa

### HIV prevention and care

Gavin George et al. (840–842) strive to improve programme effectiveness.

## Uganda

### Reaching adolescents

Sara Flanagan et al. (795–804) report trial outcomes for family planning uptake.

## Uruguay 

### COVID-19 response 

Alejandra López Gómez et al. (843–844) describe the effect of emerging evidence on public policies.

## Global

### Community health worker outcomes 

Thomas Gadsden et al. (805–818) review the evidence for performance-based incentives.

### Tailoring interventions 

Robin Schimmelpfennig et al. (819–827) promote behavioural change for health.

### Behavioural change theories

Rajiv N Rimal & Maria Knight Lapinski (828–833) describe a conceptual approach to the field.

### Behavioural and social science 

Maria A Carrasco et al. (834–836) outline research opportunities.

### Malaria control 

April Monroe et al. (837–839) study the intersection of behaviour and control efforts.

